# Sex differences in neonatal outcomes following prenatal opioid exposure

**DOI:** 10.3389/fped.2024.1357970

**Published:** 2024-03-21

**Authors:** Nethra K. Madurai, Lauren L. Jantzie, Elizabeth Yen

**Affiliations:** ^1^ Division of Neonatal-Perinatal Medicine, Department of Pediatrics, Johns Hopkins University School of Medicine, Baltimore, MD, United States; ^2^ Department of Neurodevelopmental Medicine, Phelps Center for Cerebral Palsy and Neurodevelopmental Medicine, Kennedy Krieger Institute, Baltimore, MD, United States; ^3^ Department of Neurology, Johns Hopkins University School of Medicine, Baltimore, MD, United States; ^4^ Division of Pediatric Neurosurgery, Department of Neurosurgery, Johns Hopkins University School of Medicine, Baltimore, MD, United States; ^5^ Mother Infant Research Institute (MIRI), Tufts Medical Center, Boston, MA, United States; ^6^ Division of Newborn Medicine, Tufts Medicine Pediatrics-Boston Children's, Boston, MA, United States; ^7^ Department of Pediatrics, Tufts University School of Medicine, Boston, MA, United States

**Keywords:** prenatal opioid exposure, neonatal opioid withdrawal syndrome, opioid use disorder, sex differences, newborn, development, pregnancy

## Abstract

The impact of the opioid epidemic on pregnant people and children is a growing public health crisis. Understanding how opioids affect the developing brain during pregnancy and postnatally remains a critical area of investigation. Biological sex plays a crucial role in all physiologic processes, with the potential for a significant impact on neonatal outcomes, including those infants with opioid exposure. Here, we aim to explore current literature on the effect of sex on neonatal outcomes following prenatal opioid exposure. Sex differences in adults with opioid use disorder have been well studied, including increased mortality among males and higher rates of psychiatric comorbidities and likelihood of relapse in females. However, such differences are not yet well understood in neonates. Emerging clinical data suggest sex-specific effects in infants with prenatal opioid exposure on the expression of genes related to feeding regulation and reward signaling pathways. Increased susceptibility to white matter injury has also been noted in female infants following prenatal opioid exposure. Understanding the impact of sex as a biological variable on neonatal outcomes following prenatal opioid exposure is paramount to improving the health and well-being of infants, children, and adults impacted by the opioid epidemic.

## 1 Introduction

Rates of opioid use disorder (OUD) among pregnant individuals and persons of childbearing age continue to rise (1), accompanied by a significant increase in neonatal abstinence syndrome (NAS), also known as neonatal opioid withdrawal syndrome (NOWS) (2–7). The diagnosis of NAS has risen more than five-fold from 2000 to 2016, highlighting the need for an improved understanding of risk factors associated with NAS to help inform prevention and tailor neonatal management (4, 8). Current literature suggests certain factors during pregnancy that increase the risk of NAS, including opioid type, total opioid exposure, tobacco use, and selective serotonin receptor inhibitor (SSRI) use (9). However, other factors that influence the development of NAS and its complications remain poorly understood. There has been discrepant literature regarding the impact of sex in the development of NAS and its associated complications, with some studies showing increased vulnerability among male infants (10–13), while others are more equivocal (14, 15).

**Figure 1:**
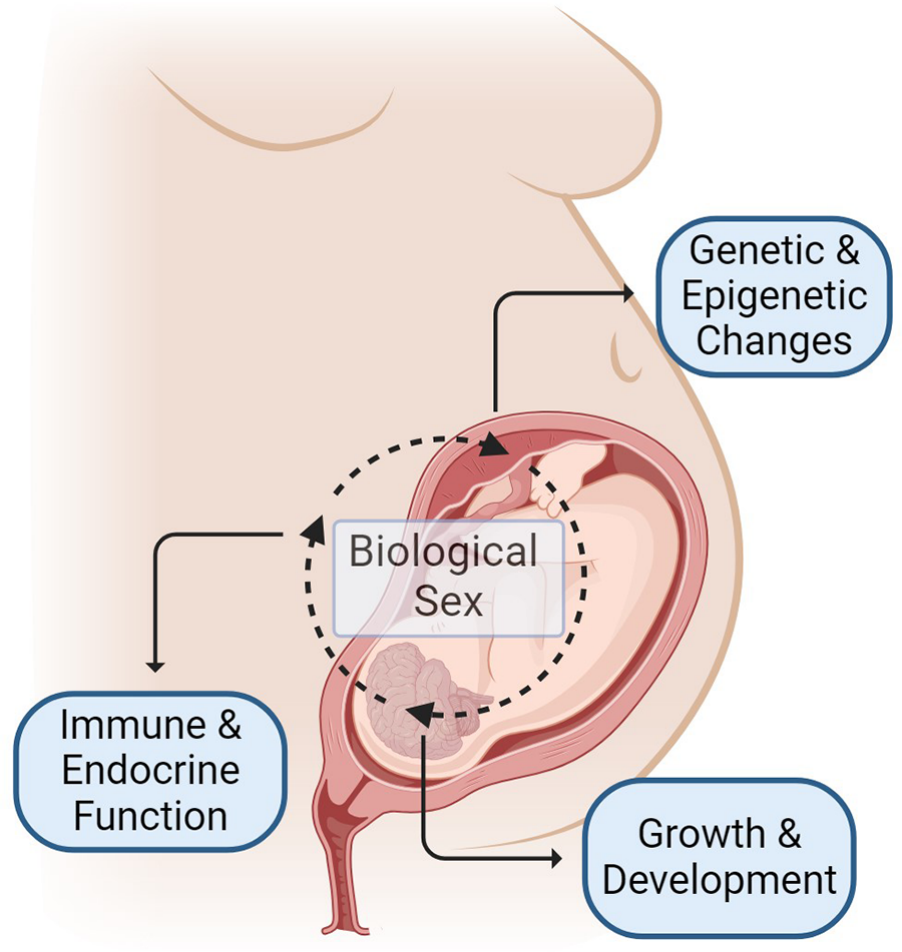
Mechanisms of sex-specific differences in outcomes following prenatal opioid exposure begins before birth and occurs due to complex interplay of many factors including, but not limited to: (1) sex-specific regulation of genetic and epigenetic changes influenced by maternal, placental and fetal factors (2) sex-specific influence on growth and neurodevelopment (3) sex-specific differences in immune and endocrine function. Figure created with BioRender.com.

Recognition of the fundamental role that sex plays in disease and health outcomes across the lifespan has grown over the previous decades (16). Sex-specific influences on the growth and development of a fetus during pregnancy start along the placental-fetal-brain axis, with salient differences noted from early in gestation (16–19) (Figure 1). Males reportedly have increased vulnerabilities in pregnancy outcomes and perinatal stressors, however, the biological mechanism underlying these differences and long-term outcomes is an active area of investigation (19–26). While opioids readily cross the placenta and blood-brain barrier in the developing fetus (27), it remains unclear how sex influences the impact of prenatal opioid exposure on infant and childhood outcomes. Here, we review preclinical and adult studies and summarize some of the proposed mechanisms for sex vulnerability in opioid-exposed neonates, including inflammation, different responses to stress, changes in the microbiome, and differences in cell death mechanisms (28–37) (Table 1).

**Table 1:** Summary of sex-specific alterations noted in endogenous and exogenous opioid functions.

Endogenous opioid effects	Exogenous opioid effects
• Neural cell specific functions: ⚬ Proper timing of neural cell maturation ⚬ Differential regulation of oligodendrocyte function and maturation based on sex	• Sex-specific influence on opioid-receptor density, microglial function, synaptic pruning pathways, and dopamine reward pathways
• Sex-specific developmental program of neuroimmune system	• Sex-specific variation in gut microbiota
• Influence of sex hormones on endogenous opioid signaling related to reproductive behaviors	• Sex-specific hypothalamic-pituitary axis dysregulation
• Sex-specific differences in endogenous opioid peptides and receptors	• Sex-specific effects on genetic and epigenetic changes in placenta and fetus

## 2 The influence of sex on opioid use and effects in offspring: preclinical and human data

### 2.1 Sex differences in preclinical data

Preclinical studies of sex as a biological variable (SABV) in prenatal opioid exposure allow for investigation in the absence of confounding variables, such as clinical and social factors, that may influence outcomes across the lifespan in humans. Alongside the establishment of the impact of sex following prenatal opioid exposure, preclinical studies also lend themselves to the exploration of the mechanisms underlying sex-specific differences.

The effects of exogenous opioid administration in the developing central nervous system (CNS) begin with an understanding of the function of the endogenous opioid system in fetal development. Hauser and Knapp highlighted the modulation of cellular proliferation and differentiation by endogenous opioids that are context-dependent and vary by cell type, timing, and duration of exposure (38). Preclinical studies have shown the expression of mu-, delta- and kappa-opioid receptors in neural precursor cells. Expression of endogenous opioid peptides and receptors allows for proper coordination and timing of proliferation and differentiation in a region-specific manner during maturation and into adulthood allowing for plasticity (38). Endogenous opioid peptides are thought to function in the normal maturation process by inhibiting the growth of neural progenitor cells and allowing sufficient time for development to occur (38). Exogenous opioid exposure during this delicate maturational process may have a significant impact on the trajectory of the developing CNS. Several preclinical studies using cell cultures and animal models to examine oligodendrocyte maturation and function with exogenous opioid exposure during pregnancy demonstrate alterations in the timing of oligodendrocyte maturation and myelination (39). These studies further support emerging clinical literature that demonstrates white matter changes in infants with prenatal opioid exposure (32, 40).

The interaction of sex on the endogenous opioid system has been further explored in the preclinical literature. In one study, cell culture models of rodent oligodendrocytes were evaluated postnatally to assess control of myelination and related processes by endogenous opioid peptide endomorphin at the mu-opioid receptor and opioid-related neuropeptide nociceptin at the nociceptin/orphanin receptor (41). The authors demonstrated an elegant interplay between endomorphin and nociceptin in regulating oligodendrocyte function and maturation restricted to those cells isolated from female pups (41). The suggestion of sex-specific effects on myelination controlled by the endogenous opioid system may have enduring ramifications for fetal brain development occurring in the context of exogenous opioid exposure.

Opioid receptor signaling and its role in regulating excitatory and inhibitory neural pathways by region is reviewed thoroughly by Reeves et al. demonstrating diversity in mechanisms of opioid-receptor mediated action (42). Hou et al. examined the impact of prenatal opioid exposure on opioid receptor signaling in mesolimbic structures in rodents during the neonatal period and found that male pups were particularly susceptible to aberrant signaling in this pathway following prenatal buprenorphine and methadone exposure (43). Altered excitatory and inhibitory synapses in the anterior cingulate cortex, nucleus accumbens, and prefrontal cortex of rodents exposed to exogenous morphine has also been noted (44). Further characterization of cell-specific roles and opioid receptor expression is necessary to gain a better understanding of the impact of endogenous and exogenous opioid signaling on brain circuitry.

Beyond neural cell-specific functions related to sex is the influence of sex-specific hormones. McCarthy et al. provided an elegant review of sex differentiation in rodent brains and behavior that stems from hormonal and chromosomal effects. Through an intricate intersection between neuroimmunity, neuroepigenetics, and neuroendocrine, sex-specific developmental programming takes place in the brain's innate immune cells, leading to unique neurodevelopment, phenotypic behaviors, and predisposition to neuropsychiatric disorders (45, 46). The effects of sex hormones are also seen in the endogenous opioid system. Estrogen and progesterone regulate endogenous opioids via mu-opioid receptor activation and expression in signaling female reproductive behaviors in several rodent models (47, 48). Similarly, androgens exert prenatal organization effects, responsible for the development of both internal and external sex organs, but more importantly, the sexual dimorphism of early brain development and subsequent behaviors throughout life (49). Sharp et al. provided a comprehensive review on sex differences in opioid-receptor mediated reward circuitry (50). Such sex differences likely determine distinct opioid-mediated dependence, sensitivity, tolerance, and withdrawal between males and females (50). Despite inconsistent findings across neuroanatomical regions and developmental stages due to complex regulatory roles inherent to the endogenous opioid system, sex-specific distinction exists in the amount and location of endogenous opioid receptors (mu-, delta-, and kappa-) and opioid peptides (beta-endorphin, met/leu-enkephalin, dynorphin). For instance, male rats have significantly greater concentrations of mu-opioid receptors in the spinal cord and midbrain than females, while females have significantly higher concentrations of kappa receptors in the spinal cord and hindbrain than males (51). Adult rat males had a greater abundance of beta endorphin in the prefrontal cortex than females, while adult females had a greater abundance of the same peptide in the hypothalamus, hippocampus, and anterior pituitary than males (52). The sex differences in endogenous opioid peptides and receptors are even more complex due to the fluctuation of these peptides and receptors over the course of the estrous cycle in females (53). Taken together, sex-specific differences in opioid receptor-mediated reward effects are complex and need to be accounted for in determining and understanding the effects of the opioid epidemic.

Preclinical literature examining the impact of exogenous opioid exposure during pregnancy on the hypothalamic-pituitary-adrenal (HPA) axis has also noted sex-specific differences in opioid-exposed adolescent and adult animals. One study demonstrates muted pituitary response to postnatal stress in adult female rodents following prenatal morphine exposure (28); another study suggests exaggerated pituitary response in adolescent males following prenatal oxycodone exposure (54). While these studies highlight sex-specific HPA dysregulation into adulthood following prenatal opioid exposure, further study of the impact of opioid type, duration of exposure, and underlying mechanisms, including receptor pathology, is needed.

There has been growing interest in the neural-gut connection and the role of the gut microbiome in pathology related to OUD (36, 37). Antione et al. studied the impact of neonatal morphine exposure in mice and found gut dysbiosis and significant alterations in gut diversity and composition. Adolescent morphine-exposed females had increased Firmicutes/Bacteroidetes (F/B) ratio compared to saline-exposed females, while no differences were noted in morphine-exposed males compared to saline-exposed males (35). These changes in morphine-exposed females persisted into adulthood (35). Chronic methadone exposure in pregnant dams also showed alterations in gut microbiota in both dams and offspring, though no sex-specific differences were found (55). The neural-gut connection and its relation to developmental pathology following prenatal opioid exposure requires further investigation.

There are limited studies in preclinical models examining the influence of sex on long-term outcomes following prenatal opioid exposure. In an animal model of prenatal methadone exposure, Grecco et al. have shown limited sex-related differences in the early postnatal period, with more pronounced sex effects on alcohol reward behaviors in adolescence (34, 56). Methadone-exposed females showed a greater preference for alcohol-associated environments, whereas methadone-exposed males demonstrated resistance to this conditioned preference (56). There were also differences in behavior responses to alcohol, with methadone-exposed adolescent females showing hyperactivity and increased locomotor activity following alcohol consumption and methadone-exposed males demonstrating more binge-like alcohol consumption (56). Interestingly, examination of mu-opioid receptor density in the reward pathway of adult rodents following prenatal morphine exposure demonstrates a significantly greater increase in mu-opioid receptor density in males compared to females (57). Morphine-exposed males were more vulnerable to executive function deficits tested in adulthood, evidenced by impaired learning, motivation, and attention (58). Further examination of sex differences in microglia and synaptic pruning pathways suggested deficits in microglial phagocytic pathways in male offspring with morphine exposure may underly these enduring behavioral impairments (58). These studies highlight the importance of understanding the developmental crosstalk between sex and prenatal opioid exposure and its influence on neuronal functioning, reward pathway signaling, and behavior into adulthood.

### 2.2 Sex differences in OUD

Paralleling the preclinical data, sex-specific effects of opioids are also evident in adults with OUD. With almost 11 million Americans reporting opioid misuse in 2016, the prevalence of OUD remains highest in males, however, the rate of use increases more rapidly in females (59). The risk of overdose is also higher in males, but emerging data highlight distinct vulnerabilities in females, especially during pregnancy and the postpartum period (60, 61).

A scoping review by Huhn et al. demonstrated several themes across the addiction cycle that were different between males and females (62). Although most studies were retrospective and not powered to assess sex differences, the authors reported a significantly higher burden of mental health disorders, particularly depression and suicidal ideation, in females compared to males at the time of presentation for treatment (62). Co-occurring mental illness, i.e., depression, in females conferred a unique risk for relapse (62). These findings underscore the need for addressing comorbid mental health conditions in treatment programs for OUD and tailoring these approaches to females. Several studies promoted the use of buprenorphine treatment for females as it was associated with lower rates of relapse and higher rates of treatment retention compared to males with OUD (62). Overall, this review identifies important themes to help inform and tailor practice strategies and highlights the need for additional prospective studies and further understanding of the biological mechanism underlying sex-related differences in the treatment of OUD. The same authors have published a systematic review of preclinical literature evaluating sex-related differences in response to endogenous and exogenous opioids, which further highlights the need for more rigorous study in this area (63).

Another study by Davis and colleagues used machine learning to assess multi-center follow-up data to evaluate sex differences in factors predicting relapse following treatment of OUD (64). Younger age at initiation and male sex were risk factors for relapse. Conduct disorder and multiple co-occurring substance use disorders posed a greater risk for return to opioid use in males (64). For females, withdrawal symptoms, depressive symptoms, and diagnosis of post-traumatic stress disorder were factors that increased the likelihood of relapse following treatment (64). Understanding sex differences in risk factors for relapse following OUD treatment may inform personalized treatment programs to mitigate such risks and, in turn, tackle the growing opioid epidemic. More rigorous clinical data from prospective studies designed to assess sex differences in OUD management and outcomes are urgently needed.

### 2.3 Sex differences in neonates with prenatal opioid exposure and clinical outcomes

NAS manifests as multiorgan withdrawal signs, e.g., high-pitch cries, tremors, hypertonia, difficulty feeding, hyperphagia, sleep disturbance, loose stools, and poor growth. Some neonates have more severe withdrawal requiring pharmacotherapy with unclear risk factors. Maternal factors (medication types, dose, comorbid physical and mental health conditions, polysubstance use, socioeconomic status), neonatal factors (gestational age, body weight), hospital-based practice variations, and genetic and epigenetic factors may contribute to specific risk profiles and predisposition to worse NAS (65–70). Opioid-exposed males and females may also exhibit different responses and clinical outcomes related to *in utero* opioid exposure. Data on sex differences in the risk, clinical presentations, and need for pharmacotherapy in opioid-exposed neonates remain unclear. Some reported no sex differences in the withdrawal severity, need for pharmacotherapy, and duration of treatment for NAS (14, 15). Others showed that male sex is a risk factor for worse withdrawal and need for pharmacotherapy, and female sex is protective against severe withdrawal (10, 11, 71, 72).

Conradt et al. showed that of the 52 studies on long-term outcomes in opioid-exposed neonates published between 1975 and 2019, only 18 were published in the last five years, pointing to the lack of longitudinal studies in the field (73). Opioid-exposed children seemed to exhibit more adverse behaviors, e.g., anxiety, aggression, fear, and have lower executive functioning, with more ambiguity in cognitive outcomes. Only two of these 18 studies were prospective, highlighting the tremendous challenge in understanding long-term outcomes in opioid-exposed neonates. Several critical methodologic shortcomings thought to contribute to such challenges were the small sample size, the abundance of confounding factors, the lack of consensus on the assessment and diagnosis of NAS, and the lack of early biomarkers to track risks and neurodevelopmental outcomes. Furthermore, less than half of these publications considered sex differences (73). Consequently, the sex-specific impact of prenatal opioid exposure remains unclear, with some reported males having poorer cognitive and language development in early childhood (74), another showing both sexes performing worse on cognitive functioning than their respective non-exposed counterparts, yet another reporting females having worse and increasing differences in cognitive at a later age (27).

Emerging studies have focused on biomarker research in NAS. Biomarker discovery has the potential to provide a mechanistic underpinning of NAS, which can inform targeted interventions and convenient monitoring for this condition (70). Existing studies demonstrated that genetic and epigenetic changes may predict withdrawal severity and the need for pharmacotherapy. These changes include variants of the cytochrome P450 family 2 subfamily B member 6 (*CYP2B6*) (75), increased methylation of ATP binding cassette subfamily B member 1 (*ABCB1*)*,* cytochrome P450 family 2 subfamily D member 6 (*CYP2D6*)*,* and opioid receptor mu 1 (*OPRM1*) (76, 77), single nucleotide polymorphism in opioid receptor kappa 1 (*OPRK1*), opioid receptor delta 1 (*OPRD1*), prepronociceptin (*PNOC*) (78), and dopamine receptor type 2 (*DRD2*) (79). The first genome-wide association studies done in opioid-exposed neonates showed a promising potential to develop a polygenic risk score that can predict the need for pharmacotherapy (80). Camerota et al. showed that pharmacotherapy for NAS decreased DNA methylation in one of four CpG sites within the *OPRM1 and was* accompanied by improved clinical presentations, e.g., reduced irritability, signs of stress, and abnormal movements (81), suggesting the utility of biomarkers in monitoring disease progression in NAS. Similar to the trend in the clinical studies, however, only a few molecular studies considered sex differences.

Sex-specific mechanisms commence long before birth and stem from an intricate gestational interplay between the mother and the fetus, between the chromosomal and hormonal effects, resulting in distinct neurobehavioral changes and sex-specific vulnerability (17, 82, 83) (Figure 1). Maternal factors (e.g., drug metabolism and immune responses) affect placental gene expression. At the same time, fetal sex also modulates placental gene transcription. Testosterone release by male fetuses in mid to late gestation differentiates the body and the brain from females, which further impacts the physiological responses and drug metabolism after birth. In addition to this hormonal effect, the sex chromosome-specific genes modulate sex-specific responses, furthering the sex-specific differences pre and postnatally (82). Converging data support the propensity of male fetuses to early-life adverse events and the greater vulnerability to subsequent developmental challenges (17, 26). Therefore, sexual dimorphism related to prenatal opioid exposure will affect the postnatal course, supporting the need to study sex differences to optimize care.

A few studies have demonstrated evidence of molecular mechanisms underlying sex-specific changes in NAS. A salivary transcriptomic study showed that opioid-exposed males had greater expression of key reward gene *DRD2* than opioid-exposed females. This sex-differential gene expression persisted with the need for pharmacotherapy and correlated positively with volume of oral intake (breastmilk, formula), suggesting sex-specific aberrant reward signaling might predispose males to worse NAS (31). Opioid-exposed females, however, are not without risks. Despite less severe overt withdrawal, exposed females had a greater incidence of white matter hyperintensity in brain imaging than exposed males (32). Such white matter hyperintensity findings were accompanied by greater expression of proinflammatory genes, suggesting sex-specific proinflammatory effects of prenatal opioids and associated brain injury with a greater effect in females than males (32). Sex-specific proinflammatory effects on the brain and feeding regulation may alter the brain-gut axis and long-term health and developmental outcomes.

Sex-specific differences in dopamine receptor gene expression implicate the disruption of mesolimbic and mesocortical dopamine pathways in neonates with prenatal opioid exposure. Postnatal neuroimaging in opioid-exposed neonates demonstrated smaller deep gray matter structures (e.g., ventrolateral thalami, subthalamic nuclei) and smaller brainstem volumes adding to the evidence of alterations in these dopaminergic pathways (84). Resting-state functional imaging demonstrates global connectivity issues in neonates with prenatal opioid exposure, suggesting the involvement of various pathways and regions in aberrations related to opioid exposure (85). These small cohort studies have controlled for sex but were not designed to show sex differences, necessitating further investigation.

Clinical and molecular studies in neonates with prenatal opioid exposure may be essential first steps to understanding sex-specific risks and vulnerability differences in adults with OUD. Prenatal opioid exposure imposes myriad health challenges, including structural and functional brain changes, cognitive and neurodevelopment, vision health, and nutrition and growth (73, 84, 86–90). Accounting for sex is crucial to understanding opioid effects and targeting efforts and interventions to prevent, cure, and ameliorate such effects. Males and females have fundamental differences that cannot be simplified or ignored, and we must meticulously study these unique characteristics to provide optimal care for this vulnerable population.

## 3 Conclusion/future directions

The present narrative review of preclinical and clinical literature reveals several salient sex-related differences in neonates, children, and adults affected by OUD and underscores the need to include SABV in future investigations. Preclinical studies with particular attention to the unique interplay between the endogenous opioid system and coordination of neural maturation and differentiation, alongside neuroendocrine effects specific to sex hormone biology, may be particularly informative in understanding the impact of prenatal opioid exposure. Future studies should focus on the sex-specific and proinflammatory impact of prenatal opioid exposure on the placenta-brain, placenta-gut, and brain-gut axes as they may elucidate novel biomarkers and treatment strategies. While there is significant biological plausibility underlying the influence of sex on prenatal opioid exposure and neonatal outcomes, a more substantial investigation into the sex-specific mechanisms underlying this developmental influence is imperative to inform the identification and management of neonates at risk for NAS. Understanding the full scope of the impact of opioid use and misuse on fetal development, neonatal, and adult outcomes is a daunting prospect, but the influence of sex in each of these pathways is an essential consideration and must not be overlooked.

## References

[1] Desai RJ Hernandez-Diaz S Bateman BT Huybrechts KF. Increase in prescription opioid use during pregnancy among medicaid-enrolled women. Obstet Gynecol. (2014) 123(5):997–1002. 10.1097/AOG.0000000000000208 24785852 PMC4020039

[2] Tolia VN Patrick SW Bennett MM Murthy K Sousa J Smith PB Increasing incidence of the neonatal abstinence syndrome in U. S. neonatal ICUs. N Engl J Med. (2015) 372(22):2118–26. 10.1056/NEJMsa1500439 25913111

[3] Winkelman TNA Villapiano N Kozhimannil KB Davis MM Patrick SW. Incidence and costs of neonatal abstinence syndrome among infants with medicaid: 2004–2014. Pediatrics. (2018) 141(4):1–8. 10.1542/peds.2017-3520 PMC5869343 29572288

[4] Hirai AH Ko JY Owens PL Stocks C Patrick SW. Neonatal abstinence syndrome and maternal opioid-related diagnoses in the US, 2010–2017. JAMA. (2021) 325(2):146. 10.1001/jama.2020.24991 33433576 PMC7804920

[5] Ko JY Patrick SW Tong VT Patel R Lind JN Barfield WD. Incidence of neonatal abstinence syndrome—28 states, 1999–2013. MMWR Morb Mortal Wkly Rep. (2016) 65(31):799–802. 10.15585/mmwr.mm6531a2 27513154

[6] Villapiano NLG Winkelman TNA Kozhimannil KB Davis MM Patrick SW. Rural and urban differences in neonatal abstinence syndrome and maternal opioid use, 2004 to 2013. JAMA Pediatr. (2017) 171(2):194. 10.1001/jamapediatrics.2016.3750 27942711

[7] Patrick SW Schumacher RE Benneyworth BD Krans EE McAllister JM Davis MM. Neonatal abstinence syndrome and associated health care expenditures. JAMA. (2012) 307(18):1934–40. 10.1001/jama.2012.3951 22546608

[8] Patrick SW Barfield WD Poindexter BB Cummings J Hand I Adams-Chapman I Neonatal opioid withdrawal syndrome. Pediatrics. (2020) 146(5):1–18. 10.1542/peds.2020-029074 33106341

[9] Patrick SW Dudley J Martin PR Harrell FE Warren MD Hartmann KE Prescription opioid epidemic and infant outcomes. Pediatrics. (2015) 135(5):842–50. 10.1542/peds.2014-3299 25869370 PMC4411781

[10] Jansson LM DiPietro JA Elko A Velez M. Infant autonomic functioning and neonatal abstinence syndrome. Drug Alcohol Depend. (2010) 109(1–3):198–204. 10.1016/j.drugalcdep.2010.01.004 20189732 PMC2875284

[11] Charles MK Cooper WO Jansson LM Dudley J Slaughter JC Patrick SW. Male sex associated with increased risk of neonatal abstinence syndrome. Hosp Pediatr. (2017) 7(6):328–34. 10.1542/hpeds.2016-0218 28465360 PMC5519405

[12] Jansson LM Dipietro JA Elko A Velez M. Maternal vagal tone change in response to methadone is associated with neonatal abstinence syndrome severity in exposed neonates. J Matern Fetal Neonatal Med. (2007) 20(9):677–85. 10.1080/14767050701490327 17701668

[13] O’Connor AB O’Brien L Alto WA. Maternal buprenorphine dose at delivery and its relationship to neonatal outcomes. Eur Addict Res. (2016) 22(3):127–30. 10.1159/000441220 26491960

[14] Holbrook A Kaltenbach K. Gender and NAS: does sex matter? Drug Alcohol Depend. (2010) 112(1–2):156–9. 10.1016/j.drugalcdep.2010.05.015 20576365

[15] Unger A Jagsch R Bäwert A Winklbaur B Rohrmeister K Martin PR Are male neonates more vulnerable to neonatal abstinence syndrome than female neonates? Gend Med. (2011) 8(6):355–64. 10.1016/j.genm.2011.10.001 22088886 PMC3241965

[16] Pinn VW. Sex and gender factors in medical studies. JAMA. (2003) 289(4):397. 10.1001/jama.289.4.397 12533102

[17] DiPietro JA Voegtline KM. The gestational foundation of sex differences in development and vulnerability. Neuroscience. (2017) 342:4–20. 10.1016/j.neuroscience.2015.07.068 26232714 PMC4732938

[18] Bake S Rouzer SK Mavuri S Miranda RC Mahnke AH. The interaction of genetic sex and prenatal alcohol exposure on health across the lifespan. Front Neuroendocrinol. (2023) 71:101103. 10.1016/j.yfrne.2023.101103 37802472 PMC10922031

[19] Kirchengast S Hartmann B. The male disadvantage hypothesis reconsidered: is there really a weaker sex? An analysis of gender differences in newborn somatometrics and vital parameters. J Life Sci. (2009) 1(1):63–71. .24611315

[20] Bekedam DJ Engelsbel S Mol BWJ Buitendijk SE van der Pal-de Bruin KM. Male predominance in fetal distress during labor. Am J Obstet Gynecol. (2002) 187(6):1605–7. 10.1067/mob.2002.127379 12501071

[21] Sheiner E Hadar A Hallak M Katz M Mazor M Shoham-Vardi I. Clinical significance of fetal heart rate tracings during the second stage of labor. Obstet Gynecol. (2001) 97(5):747–52. 10.1016/s0029-7844(01)01188-7 11339928

[22] Nagy E Loveland KA Orvos H Molnár P. Gender-related physiologic differences in human neonates and the greater vulnerability of males to developmental brain disorders. J Gend Specif Med. (2001) 4(1):41–9. . 11324239

[23] Weinberg MK Tronick EZ Cohn JF Olson KL. Gender differences in emotional expressivity and self-regulation during early infancy. Dev Psychol. (1999) 35(1):175–88. 10.1037/0012-1649.35.1.175 9923473

[24] Eogan MA. Effect of fetal sex on labour and delivery: retrospective review. Br Med J. (2003) 326(7381):137–137. 10.1136/bmj.326.7381.137 12531846 PMC140006

[25] Ingemarsson I. Gender aspects of preterm birth. BJOG. (2003) 110:34–8. 10.1046/j.1471-0528.2003.00022.x 12763109

[26] Chalak LF Pruszynski JE Spong CY. Sex vulnerabilities to hypoxia-ischemia at birth. JAMA Netw Open. (2023) 6(8):e2326542. 10.1001/jamanetworkopen.2023.26542 37526938 PMC10394577

[27] Skumlien M Ibsen IO Kesmodel US Nygaard E. Sex differences in early cognitive development after prenatal exposure to opioids. J Pediatr Psychol. (2020) 45(5):475–85. 10.1093/jpepsy/jsaa008 32324876 PMC7233842

[28] Šlamberová R Rimanóczy Á Riley MA Vathy I. Hypothalamo-pituitary-adrenal axis-regulated stress response and negative feedback sensitivity is altered by prenatal morphine exposure in adult female rats. Neuroendocrinology. (2004) 80(3):192–200. 10.1159/000082359 15583475

[29] Madurai NK Kitase Y Hamimi S Kirk SE Sevensky R Ramachandra S Methadone alters the peripheral inflammatory and central immune landscape following prenatal exposure in rats. Adv Drug Alcohol Res. (2022) 2:1–13. 10.3389/adar.2022.10792 PMC10312988 37396628

[30] Jantzie LL Maxwell JR Newville JC Yellowhair TR Kitase Y Madurai N Prenatal opioid exposure: the next neonatal neuroinflammatory disease. Brain Behav Immun. (2020) 84:45–58. 10.1016/j.bbi.2019.11.007 31765790 PMC7010550

[31] Yen E Kaneko-Tarui T Ruthazer R Harvey-Wilkes K Hassaneen M Maron JL. Sex-dependent gene expression in infants with neonatal opioid withdrawal syndrome. J Pediatr. (2019) 214:60–5.e2. 10.1016/j.jpeds.2019.07.032 31474426 PMC10564583

[32] Yen E Madan N Tarui T Kaneko-Tarui T Breeze JL Davis JM Sex-specific inflammatory and white matter effects of prenatal opioid exposure: a pilot study. Pediatr Res. (2023) 93(3):604–11. 10.1038/s41390-022-02357-5 36280708 PMC9998341

[33] Grecco GG Huang JY Muñoz B Doud EH Hines CD Gao Y Sex-dependent synaptic remodeling of the somatosensory Cortex in mice with prenatal methadone exposure. Adv Drug Alcohol Res. (2022) 2:1–22. 10.3389/adar.2022.10400 PMC10569410 37829495

[34] Grecco GG Mork BE Huang JY Metzger CE Haggerty DL Reeves KC Prenatal methadone exposure disrupts behavioral development and alters motor neuron intrinsic properties and local circuitry. eLife. (2021) 10:1–29. 10.7554/eLife.66230 PMC7993998 33724184

[35] Antoine D Singh PK Tao J Roy S. Neonatal morphine results in long-lasting alterations to the gut microbiome in adolescence and adulthood in a murine model. Pharmaceutics. (2022) 14(9):1879. 10.3390/pharmaceutics14091879 36145627 PMC9503694

[36] Abu Y Tao J Dutta R Yan Y Vitari N Kolli U Brief hydromorphone exposure during pregnancy sufficient to induce maternal and neonatal microbial dysbiosis. J Neuroimmune Pharmacol. (2022) 17(1–2):367–75. 10.1007/s11481-021-10019-2 34562195 PMC10117152

[37] Jalodia R Abu YF Oppenheimer MR Herlihy B Meng J Chupikova I Opioid use, gut dysbiosis, inflammation, and the nervous system. J Neuroimmune Pharmacol. (2022) 17(1–2):76–93. 10.1007/s11481-021-10046-z 34993905 PMC10276355

[38] Hauser KF Knapp PE. Opiate drugs with abuse liability hijack the endogenous opioid system to disrupt neuronal and glial maturation in the central nervous system. Front Pediatr. (2018) 5:1–23. 10.3389/fped.2017.00294 PMC5787058 29410949

[39] Velasco B Mohamed E Sato-Bigbee C. Endogenous and exogenous opioid effects on oligodendrocyte biology and developmental brain myelination. Neurotoxicol Teratol. (2021) 86:107002. 10.1016/j.ntt.2021.107002 34126203 PMC8277740

[40] Merhar SL Parikh NA Braimah A Poindexter BB Tkach J Kline-Fath B. White matter injury and structural anomalies in infants with prenatal opioid exposue. Am J Neuroradiol. (2019) 40:2161–5. 10.3174/ajnr.A6282 31624119 PMC6911627

[41] Mohamed E Paisley CE Meyer LC Bigbee JW Sato-Bigbee C. Endogenous opioid peptides and brain development: endomorphin-1 and nociceptin play a sex-specific role in the control of oligodendrocyte maturation and brain myelination. Glia. (2020) 68(7):1513–30. 10.1002/glia.23799 32065429 PMC11006003

[42] Reeves KC Shah N Muñoz B Atwood BK. Opioid receptor-mediated regulation of neurotransmission in the brain. Front Mol Neurosci. (2022) 15:1–28. 10.3389/fnmol.2022.919773 PMC9242007 35782382

[43] Hou Y Tan Y Belcheva MM Clark AL Zahm DS Coscia CJ. Differential effects of gestational buprenorphine, naloxone, and methadone on mesolimbic *μ* opioid and ORL1 receptor G protein coupling. Dev Brain Res. (2004) 151(1–2):149–57. 10.1016/j.devbrainres.2004.05.002 15246701

[44] Boggess T Williamson JC Niebergall EB Sexton H Mazur A Egleton RD Alterations in excitatory and inhibitory synaptic development within the mesolimbic dopamine pathway in a mouse model of prenatal drug exposure. Front Pediatr. (2021) 9:1–14. 10.3389/fped.2021.794544 PMC8710665 34966707

[45] McCarthy MM Nugent BM Lenz KM. Neuroimmunology and neuroepigenetics in the establishment of sex differences in the brain. Nat Rev Neurosci. (2017) 18(8):471–84. 10.1038/nrn.2017.61 28638119 PMC5771241

[46] Arambula SE McCarthy MM. Neuroendocrine-immune crosstalk shapes sex-specific brain development. Endocrinology. (2020) 161(6):1–13. 10.1210/endocr/bqaa055 PMC7217281 32270188

[47] Sinchak K Micevych PE. Progesterone blockade of estrogen activation of μ-opioid receptors regulates reproductive behavior. J Neurosci. (2001) 21(15):5723–9. 10.1523/JNEUROSCI.21-15-05723.2001 11466444 PMC6762652

[48] Micevych PE Rissman EF Gustafsson J Sinchak K. Estrogen receptor-*α* is required for estrogen-induced μ-opioid receptor internalization. J Neurosci Res. (2003) 71(6):802–10. 10.1002/jnr.10526 12605406

[49] Sato SM Schulz KM Sisk CL Wood RI. Adolescents and androgens, receptors and rewards. Horm Behav. (2008) 53(5):647–58. 10.1016/j.yhbeh.2008.01.010 18343381 PMC2435368

[50] Sharp JL Pearson T Smith MA. Sex differences in opioid receptor mediated effects: role of androgens. Neurosci Biobehav Rev. (2022) 134:104522. 10.1016/j.neubiorev.2022.104522 34995646 PMC8872632

[51] Bernal SA Morgan MM Craft RM. PAG Mu opioid receptor activation underlies sex differences in morphine antinociception. Behav Brain Res. (2007) 177(1):126–33. 10.1016/j.bbr.2006.10.028 17118467 PMC1868665

[52] Pluchino N Ninni F Casarosa E Giannini A Merlini S Cubeddu A Sex differences in brain and plasma beta-endorphin content following testosterone, dihydrotestosterone and estradiol administration to gonadectomized rats. Neuroendocrinology. (2009) 89(4):411–23. 10.1159/000209506 19295188

[53] Bradshaw H Miller J Ling Q Malsnee K Ruda MA. Sex differences and phases of the estrous cycle alter the response of spinal cord dynorphin neurons to peripheral inflammation and hyperalgesia. Pain. (2000) 85(1–2):93–9. 10.1016/S0304-3959(99)00253-5 10692607

[54] Sithisarn T Bada HS Dai H Reinhardt CR Randall DC Legan SJ. Effects of perinatal oxycodone exposure on the response to CRH in late adolescent rats. Neurotoxicol Teratol. (2008) 30(2):118–24. 10.1016/j.ntt.2007.12.010 18255259

[55] Grecco GG Gao Y Gao H Liu Y Atwood BK. Prenatal opioid administration induces shared alterations to the maternal and offspring gut microbiome: a preliminary analysis. Drug Alcohol Depend. (2021) 227:108914. 10.1016/j.drugalcdep.2021.108914 34364194 PMC8464518

[56] Grecco GG Haggerty DL Reeves KC Gao Y Maulucci D Atwood BK. Prenatal opioid exposure reprograms the behavioural response to future alcohol reward. Addict Biol. (2022) 27(2):1–21. 10.1111/adb.13136 PMC8896285 35229956

[57] Vathy I Šlamberová R Rimanóczy Á Riley MA Bar N. Autoradiographic evidence that prenatal morphine exposure sex-dependently alters μ-opioid receptor densities in brain regions that are involved in the control of drug abuse and other motivated behaviors. Prog Neuropsychopharmacol Biol Psychiatry. (2003) 27(3):381–93. 10.1016/S0278-5846(02)00355-X 12691773

[58] Smith BL Guzman TA Brendle AH Laaker CJ Ford A Hiltz AR Perinatal morphine exposure leads to sex-dependent executive function deficits and microglial changes in mice. eNeuro. (2022) 9(5):ENEURO.0238-22.2022. 10.1523/ENEURO.0238-22.2022 36216505 PMC9581576

[59] Volkow ND Jones EB Einstein EB Wargo EM. Prevention and treatment of opioid misuse and addiction. JAMA Psychiatry. (2019) 76(2):208. 10.1001/jamapsychiatry.2018.3126 30516809

[60] Blanco C Volkow ND. Management of opioid use disorder in the USA: present status and future directions. Lancet. (2019) 393(10182):1760–72. 10.1016/S0140-6736(18)33078-2 30878228

[61] Han B Compton WM Einstein EB Elder E Volkow ND. Pregnancy and postpartum drug overdose deaths in the US before and during the COVID-19 pandemic. JAMA Psychiatry. (2023) 81(3):270–83. 10.1001/jamapsychiatry.2023.4523 PMC10918496 37991773

[62] Huhn AS Berry MS Dunn KE. Review: sex-based differences in treatment outcomes for persons with opioid use disorder. Am J Addict. (2019) 28(4):246–61. 10.1111/ajad.12921 31131505 PMC6591072

[63] Huhn AS Berry MS Dunn KE. Systematic review of sex-based differences in opioid-based effects. Int Rev Psychiatry. (2018) 30(5):107–16. 10.1080/09540261.2018.1514295 30522374 PMC6551331

[64] Davis JP Eddie D Prindle J Dworkin ER Christie NC Saba S Sex differences in factors predicting post-treatment opioid use. Addiction. (2021) 116(8):2116–26. 10.1111/add.15396 33405314 PMC8254742

[65] Minozzi S Amato L Jahanfar S Bellisario C Ferri M Davoli M. Maintenance agonist treatments for opiate-dependent pregnant women. Cochrane Database Syst Rev. (2020) 2020(11):1–20. 10.1002/14651858.CD012557.pub2 PMC8094273 33165953

[66] Jansson LM Velez ML McConnell K Spencer N Tuten M Jones H Maternal buprenorphine treatment and infant outcome. Drug Alcohol Depend. (2017) 180:56–61. 10.1016/j.drugalcdep.2017.08.001 28869859 PMC5788458

[67] Favara MT Carola D Jensen E Cook A Genen L Dysart K Maternal breast milk feeding and length of treatment in infants with neonatal abstinence syndrome. J Perinatol. (2019) 39(6):876–82. 10.1038/s41372-019-0374-1 30988400

[68] Bogen DL Whalen BL Kair LR Vining M King BA. Wide variation found in care of opioid-exposed newborns. Acad Pediatr. (2017) 17(4):374–80. 10.1016/j.acap.2016.10.003 27889436 PMC5420467

[69] Krans EE Kim JY Chen Q Rothenberger SD James AE Kelley D Outcomes associated with the use of medications for opioid use disorder during pregnancy. Addiction. (2021) 116(12):3504–14. 10.1111/add.15582 34033170 PMC8578145

[70] Yen E Gaddis N Jantzie L Davis JM. A review of the genomics of neonatal abstinence syndrome. Front Genet. (2023) 14:1–8. 10.3389/fgene.2023.1140400 PMC9950123 36845389

[71] O’Connor AB O’Brien L Alto WA. Are there gender related differences in neonatal abstinence syndrome following exposure to buprenorphine during pregnancy? JPME. (2013) 41(5):621–3. 10.1515/jpm-2012-0288 23612625

[72] Patrick SW Slaughter JC Harrell FE Martin PR Hartmann K Dudley J Development and validation of a model to predict neonatal abstinence syndrome. J Pediatr. (2021) 229:154–60.e6. 10.1016/j.jpeds.2020.10.030 33080277 PMC7855864

[73] Conradt E Flannery T Aschner JL Annett RD Croen LA Duarte CS Prenatal opioid exposure: neurodevelopmental consequences and future research priorities. Pediatrics. (2019) 144(3):1–25. 10.1542/peds.2019-0128 PMC6759228 31462446

[74] Nygaard E Moe V Slinning K Walhovd KB. Longitudinal cognitive development of children born to mothers with opioid and polysubstance use. Pediatr Res. (2015) 78(3):330–5. 10.1038/pr.2015.95 25978800 PMC4539602

[75] Mactier H McLaughlin P Gillis C Osselton M. Variations in infant CYP2B6 genotype associated with the need for pharmacological treatment for neonatal abstinence syndrome in infants of methadone-maintained opioid-dependent mothers. Am J Perinatol. (2017) 34(09):918–21. 10.1055/s-0037-1600917 28320034

[76] McLaughlin P Mactier H Gillis C Hickish T Parker A Liang WJ Increased DNA methylation of ABCB1, CYP2D6, and OPRM1 genes in newborn infants of methadone-maintained opioid-dependent mothers. J Pediatr. (2017) 190:180–4.e1. 10.1016/j.jpeds.2017.07.026 28867064

[77] Wachman EM Hayes MJ Shrestha H Nikita FNU Nolin A Hoyo L Epigenetic variation in *OPRM1* gene in opioid-exposed mother-infant dyads. Genes Brain Behav. (2018) 17(7):1–7. 10.1111/gbb.12476 29575474

[78] Wachman EM Hayes MJ Sherva R Brown MS Davis JM Farrer LA Variations in opioid receptor genes in neonatal abstinence syndrome. Drug Alcohol Depend. (2015) 155:253–9. 10.1016/j.drugalcdep.2015.07.001 26233486 PMC4581974

[79] Oei JL Xu HX Abdel-Latif ME Vunnam K Al-Amry A Clews S Dopamine D2 receptor gene polymorphisms in newborn infants of drug-using women. Arch Dis Child Fetal Neonatal Ed. (2012) 97(3):F193–8. 10.1136/archdischild-2011-300235 21948329

[80] Bibi S Gaddis N Johnson EO Lester BM Kraft W Singh R Polygenic risk scores and the need for pharmacotherapy in neonatal abstinence syndrome. Pediatr Res. (2023) 93(5):1368–74. 10.1038/s41390-022-02243-0 35974158 PMC9931940

[81] Camerota M Davis JM Dansereau LM Oliveira EL Padbury JF Lester BM. Effects of pharmacologic treatment for neonatal abstinence syndrome on DNA methylation and neurobehavior: a prospective cohort study. J Pediatr. (2022) 243:21–6. 10.1016/j.jpeds.2021.12.057 34971656 PMC8960328

[82] Terasaki LS Gomez J Schwarz JM. An examination of sex differences in the effects of early-life opiate and alcohol exposure. Philos Trans R Soc, B. (2016) 371(1688):20150123. 10.1098/rstb.2015.0123 PMC4785906 26833841

[83] DiPietro JA Costigan KA Voegtline KM. Studies in fetal behavior: revisited, renewed, and reimagined. Monogr Soc Res Child Dev. (2015) 80(3):vii. 10.1111/mono.12170 26303396 PMC4835043

[84] Merhar SL Kline JE Braimah A Kline-Fath BM Tkach JA Altaye M Prenatal opioid exposure is associated with smaller brain volumes in multiple regions. Pediatr Res. (2021) 90(2):397–402. 10.1038/s41390-020-01265-w 33177677 PMC8110593

[85] Radhakrishnan R Vishnubhotla RV Zhao Y Yan J He B Steinhardt N Global brain functional network connectivity in infants with prenatal opioid exposure. Front Pediatr. (2022) 10:1–10. 10.3389/fped.2022.847037 PMC8964084 35359894

[86] Radhakrishnan R Elsaid NMH Sadhasivam S Reher TA Hines AC Yoder KK Resting state functional MRI in infants with prenatal opioid exposure-a pilot study. Neuroradiology. (2021) 63(4):585–91. 10.1007/s00234-020-02552-3 32978671 PMC9162800

[87] Uebel H Wright IM Burns L Hilder L Bajuk B Breen C Reasons for rehospitalization in children who had neonatal abstinence syndrome. Pediatrics. (2015) 136(4):e811–20. 10.1542/peds.2014-2767 26371197

[88] Corr TE Schaefer EW Paul IM. Body composition during the first 4 months in infants affected by neonatal abstinence syndrome: a pilot study. J Dev Orig Health Dis. (2022) 13(1):120–7. 10.1017/S2040174421000052 33650484

[89] Vance J Chant D Tudehope D Gray P Hayes A. Infants born to narcotic dependent mothers: physical growth patterns in the first 12 months of life. J Paediatr Child Health. (1997) 33(6):504–8. 10.1111/j.1440-1754.1997.tb01659.x 9484681

[90] LaGasse LL Gaskins RB Bada HS Shankaran S Liu J Lester BM Prenatal cocaine exposure and childhood obesity at nine years. Neurotoxicol Teratol. (2011) 33(2):188–97. 10.1016/j.ntt.2010.11.002 21109003 PMC3058125

